# How pure are your vesicles?

**DOI:** 10.3402/jev.v2i0.19861

**Published:** 2013-01-10

**Authors:** Jason Webber, Aled Clayton

**Affiliations:** Institute of Cancer and Genetics, School of Medicine, Velindre Cancer Centre, Cardiff University, Cardiff, UK

**Keywords:** extracellular vesicles, nano-particle tracking, vesicle counting, sample purity

## Abstract

We propose a straightforward method to estimate the purity of vesicle preparations by comparing the ratio of nano-vesicle counts to protein concentration, using tools such as the increasingly available NanoSight platform and a colorimetric protein assay such as the BCA-assay. Such an approach is simple enough to apply to every vesicle preparation within a given laboratory, assisting researchers as a routine quality control step. Also, the approach may aid in comparing/standardising vesicle purity across diverse studies, and may be of particular importance in evaluating vesicular biomarkers. We herein propose some criteria to aid in the definition of pure vesicles.

Various biological fluids have been shown to contain extracellular vesicles. The utility of extracellular vesicles as disease biomarkers has attracted considerable interest in recent years (1). Studies to evaluate the physiological role(s) of extracellular vesicles also continue to rapidly advance the field (2), and recently their manipulation as therapeutic agents is now being vigorously investigated (3). However, there is a fairly fundamental issue in these research areas that is not satisfactorily addressed, i.e. how pure are the vesicle preparations being analysed.

Most researchers utilise ultracentrifugation-based protocols for vesicle purifications (4), but this approach can co-isolate a complex assortment of non-vesicular materials. This is a particular problem with challenging source material such as serum, urine or cancer-related effusions, estimating the purity of samples remains difficult, with inconsistent approaches across diverse studies.

Knowing something about purity is of critical importance to demonstrate, for example, that a given biomarker or functional property is associated with vesicles and not with co-isolated contaminants. There are several current approaches that attempt to address this. First, examination of samples by electron microscopy can be informative, giving an indication of vesicular morphology and revealing the presence of larger non-vesicular particulates. However, this approach cannot measure the amount of soluble factors contaminating the sample. It is also a method that is unsuited to routine daily use, as not every research group has ready access to EM.

Another approach is to look specifically for proteins that would not be expected in a vesicle preparation. Western blotting for markers such as calnexin or gp96 is commonly utilised for this purpose. Whilst this approach can be informative, it is not quantitative, and the selection of these “exclusion markers” is difficult. For example, some complement components (5) or IgG (6) may be genuinely associated with some exosomes and assuming their presence within vesicles preparations as contaminants may not be strictly accurate. Furthermore, our incomplete understanding of how proteins are loaded into vesicles, and how strictly controlled this may be under varying situations such as in disease, compounds this approach significantly.

Being able to estimate and compare sample purity, in a general, simple and quantitative manner will be immensely useful. Be it from an intra-group perspective as a general tool to monitor the quality of preparations, or more broadly as an approach to aid in establishing some international standardisation in the field as to what is an acceptably pure vesicular sample. This aspect will be of particular relevance with the advent of exosome therapeutics in humans.

Here, we present an approach that appears to serve this requirement well, based simply on measuring the particle to protein ratio.

## Methods

### Source of exosomes

Cancer cell lines were maintained at high cell density in Integra bioreactor flasks (7) or, where stated, in standard 2D 75 cm^2^ flasks. Cell lines included DU145, LNCAP, PC3 (prostate cancer), HT1376 (bladder cancer) and MCF7 (breast cancer), all from ATCC, Teddington, UK. We also used a mesothelioma cell line, developed in the department from pleural fluid specimens (we term #15). Cells were maintained in RPMI1640, with L-glutamine and antibiotics, and 10% FBS (Lonza). The FBS was depleted of vesicles by overnight ultracentrifugation at 100,000 g, followed by filtration through 0.22 and then 0.1-µm vacuum-driven filter (Millipore). As a source of *ex vivo* exosomes, we collected fresh urine from three healthy male volunteers and fresh serum from three healthy donors.

### Exosome isolation

Exosomes were purified from cell-conditioned media (10–15 ml) or biological fluids (urine>10 ml; serum <2 ml), using a basic differential ultracentrifugation method (400 g, 5 min, 2,000 g, 15 min, 10,000 g, 40 min), followed by centrifugation at 100,000 g for 60 min. For some isolations, this final pellet was subjected to a single wash step by re-suspending in 5 ml PBS and centrifuging again at 100,000 g, 60 min (Optima-MAX ultracentrifuge, with TLA110 rotor and Optiseal tubes, Beckman coulter). For cells cultured in bioreactor flasks, we employed our usual exosome isolation method, involving pre-clearing centrifugations as above, but substituting the first pelleting step with centrifugation on a 30% sucrose/D_2_O cushion for 60 min. The collected cushion was subjected to one wash in PBS (8). For urine and serum samples, specimens were subjected to the same pre-clearing steps, but the pelleting speed used was 120,000 g.

### Protein assay

An aliquot of each preparation was kept for protein estimation using the micro-BCA kit from Thermo Scientific Pierce (Thermo Fisher Scientific, Northumberland, UK). Exosome preparations, usually diluted 1 in 8 to 1 in 20, were compared in triplicates against serially diluted BSA as standard. Values were extrapolated from this curve, using a third-order polynomial equation, with *r*^2^>0.98 for each assay.

### Nanoparticle tracking analysis (NanoSight*™*)

Vesicles present in purified or unpurified samples were analysed by nanoparticle tracking, using the NanoSight LM10 system (NanoSight Ltd, Amesbury, UK), configured with a 405 nm laser and a high sensitivity digital camera system (OrcaFlash2.8, Hamamatsu C11440, NanoSight Ltd). Videos were collected and analysed using the NTA-software (version 2.3), with the minimal expected particle size, minimum track length, and blur setting, all set to automatic. Camera shutter speed was fixed at 30.01 ms and camera gain was set to 500. Camera sensitivity and detection threshold were set close to maximum (15 or 16) and minimum (3 or 4), respectively, to reveal small particles. Ambient temperature was recorded manually, ranging from 24 to 27°C. Each sample was diluted in nanoparticle-free water (Fresenius Kabi, Runcorn, UK), so that the concentration was between 2×10^8^ and 9×10^8^ particles/ml. Samples were administered and recorded under controlled flow, using the NanoSight syringe pump and script control system, and for each sample, six videos of 30–60 seconds duration were recorded, with a 10-second delay between recordings, generating six replicate histograms that were averaged. Therefore, the typical number of completed tracks per sample was approximately 1,200. The area under the curve was calculated using Prism-4 software version 4.03 (Graph Pad, San Diego, CA), to give average particle counts from these replicates.

### Statistical analysis

Graphs and statistical analyses were performed using Prism-4 software (version 4.03, Graph Pad, San Diego, CA). In all experiments, one-way ANOVA, with Tukey's post-test was used. Differences with *p* values of 0.05 or less are considered significant *p<0.05, **p<0.001, ***p<0.0001.

## Results

### Particle counting by NanoSight

**Fig. 1:**
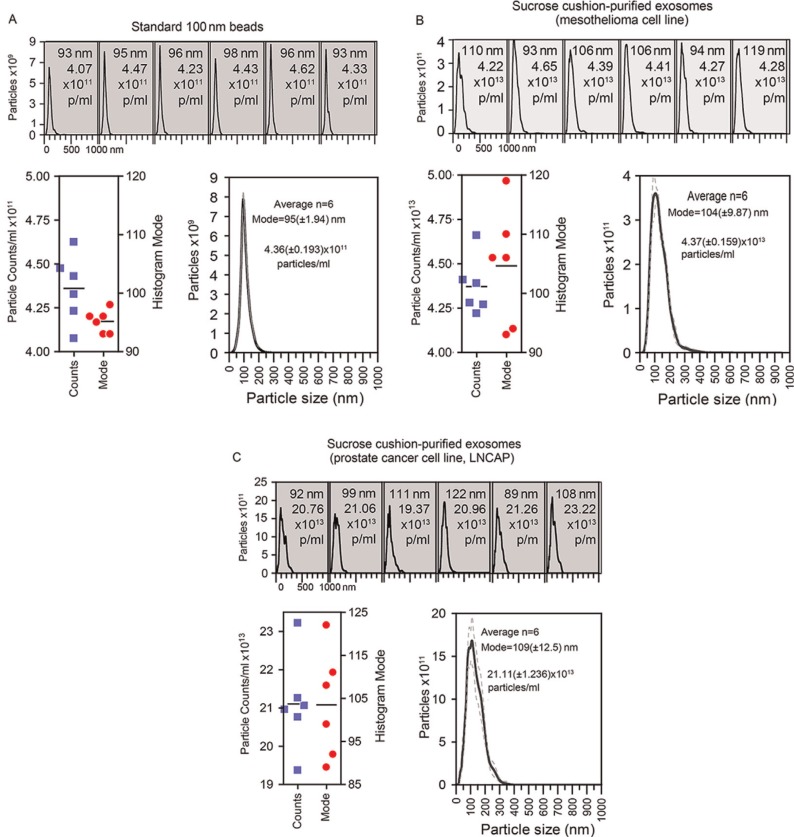
Measuring beads or extracellular vesicles under flow conditions by nanoparticle tracking. (A) Standard 100 nm beads were diluted (1 in 1,000) in particle-free water, and measured six times using the NanoSight nanoparticle tracking system. Data from each repeat measurement is shown, revealing the overall size distribution (histograms) and mode (nm) and particle counts (p/ml). To evaluate reproducibility of the measurements, the counts (blue squares) and mode (red circles) for each measurement is shown. An average histogram was plotted from the data, and the mode and particle concentration is calculated (±SD). (B, C) As examples of biological vesicles, similar analyses using sucrose cushion isolated exosomes secreted from a mesothelioma cell line (B) or the prostate cancer cell line, LNCAP, (C) showing each measurement and the variation across the six measurements.

As an example of nanoparticle analysis, we present typical analyses of standard nano-beads (of 100 nm size), measured under fluid flow six times (Fig. 1A). Each individual histogram is shown, and this is annotated with the histogram mode and total particle concentration. To reveal the variation across the measurements, these data are plotted as individual points, and the average of these measurements is also shown. This reveals a mode of 95±1.94 nm for the averaged histogram, which sits within the expected range of size discrimination of the instrument, and compares with other NanoSight-based observations (9).

In contrast, Fig. 1B reveals analysis of sucrose-cushion-purified exosomes, from a mesothelioma cell line, or from the LNCAP cell line (Fig. 1C) performed in an identical manner. However, this more complex sample reveals greater variation in the histogram mode compared to the 100 nm standard beads, and this justifies our choice to run multiple measurements of each sample. The variation seen in particle counts is also shown. We have previously documented the molecular phenotype and structure of exosomes isolated using the sucrose cushion approach, by western blotting, electron microscopy and other methods (8, 10,11) revealing these as quality preparations according to generally accepted criteria.

All particle counting data that follow in this manuscript were performed in this manner.

### Protein contamination and ratio measurements

**Fig. 2:**
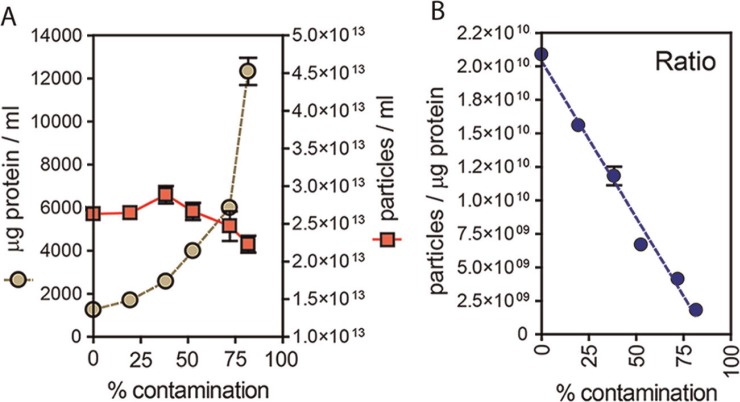
Particle to protein ratio diminishes with contaminating protein. (A) Extracellular vesicles were purified from prostate cancer cell line (LNCAP) using the sucrose cushion method, and were intentionally contaminated with a solution of bovine serum albumin that was pre-filtered through a 20 nm filter, and confirmed particle free (not shown). The graph plots the protein concentration (µg/ml, left axis) and particle concentration (particles/ml, right axis) against the proportion of non-exosomal (contaminating) protein (% contamination). (B) The ratio of particles to protein for each sample is shown.

We hypothesise that vesicle preparations that are pure exhibit a relatively high ratio of particles to protein and thus introducing contaminating protein to the samples should have a negative effect on the ratio. To empirically test this, we took a typical sucrose-cushion purified exosome preparation, from the LNCAP cell line, and added elevating concentrations of exogenous BSA. We took care to filter this BSA through a 20 nm filter and confirmed this was particle-free using the NanoSight system (not shown). At each dose of BSA, a protein assay was performed, and nanoparticles counted as described. The data show that as protein concentration escalates (Fig. 2A circles), this has little impact on nanoparticle counts (Fig. 2A squares). Plotting the ratio of particles per µg of protein (Fig. 2B), demonstrates falling ratios correlate with samples of decreasing purity, with a 50% decrease in ratio approximately equating to an increase in non-vesicular protein of approximately 40–50%, in this assay.

### Ratio of particle to protein as a means of comparing sample purity

**Fig. 3:**
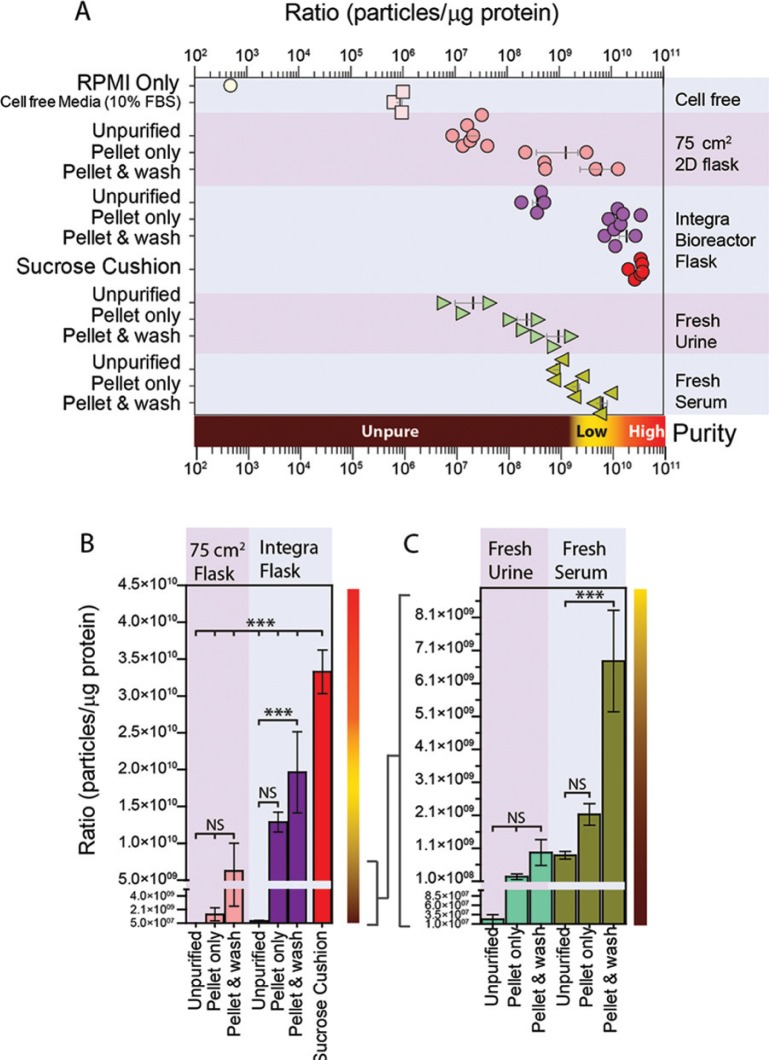
Use of particle to protein ratio to quantify vesicle purity. (A) We compared the ratio of particles/protein across various sample types, and purification methods. This includes specimens from standard 2D plastic-adherent cultures including un-purified particles (MCF7 *n*=3, PC3 *n*=2, DU145 *n*=2) vs. simple pellet (MCF7 *n*=1, PC3 *n*=1, DU145 *n*=1) vs. pellet and wash (MCF7 *n*=1, PC3 *n*=1, DU145 *n*=1) methods. These are compared to un-purified particles (PC3 *n*=1, DU145 *n*=2, HT1376 *n*=1) vs. simple pellet (PC3 *n*=1, Du145 *n*=1, Ht1376 *n*=1, LNCAP *n*=1, #15 *n*=1) vs. pellet and wash (PC3 *n*=1, Du145 *n*=1, Ht1376 *n*=1, LNCAP *n*=1, #15 *n*=1) specimens obtained from Integra bioreactor culture systems, and biological specimens such as fresh serum (*n*=3) and urine samples (*n*=3). A sucrose cushion method was used with Integra bioreactor culture supernatants (PC3 *n*=1, Du145 *n*=2, Ht1376 *n*=1, LNCAP *n*=1, #15 *n*=1). The ratio measurement for RPMI and cell-free RPMI containing 10% FBS is also shown for comparison. (B/C) The same data are presented as linear plots, to better highlight the difference in ratio due to pellet and pellet and wash methods. From the collective data, ratios approaching 3×10^10^ are highlighted as high purity, with those <10^8^ are arbitrarily considered unpure.

Cell-conditioned media was collected from various cultured cell lines, maintained either in the usual 2D 75 cm^2^ flasks (Fig. 3, pink circles) or in Integra bioreactor flasks (Fig. 3, purple and red circles). For both cell culture systems, ratios were calculated for un-purified conditioned media, or following pelleting or pellet and wash purification methods. For the Integra bioreactor-derived media, we also conducted our usual sucrose cushion protocol (Fig. 3, red circles). Cultures from 2D 75 cm^2^ flasks gave a ratio of approximately 1×10^7^ particles per µg protein (P/µg), and this was elevated 60-fold by pelleting and a further 4.6-fold after a PBS wash. Media from Integra bioreactors has higher concentrations of exosomes, but a comparable amount of non-vesicular material from FBS, hence these ratios were higher at 3.7×10^8^ P/µg, and pelleting and washing elevated the ratio to around 2×10^10^ P/µg. Using the sucrose cushion method with this starting material, however, gave superior ratios approaching 3.4×10^10^ P/µg. For completeness, we also examined serum-free RPMI, and solutions of 10% FBS in RPMI, using FBS that had apparently been depleted of exosomes as described in the methods. In the virtual absence of particles, the ratio for RPMI was negligible, but there remained detectable particles within the 10% FBS/RPMI (ratio of 1×10^6^ P/µg), defining this as the background level of non-cell-derived particles in our culture-derived samples.

Analyses using fresh urine or serum revealed similar findings, with elevating ratios following pelleting and washing. However, using these protocols, it was clear that the ratios achievable using such complex source material remain vastly inferior to that of cell culture sources. These data in totum are summarised in Fig. 3A (logarithmic scale), and also presented in linear scale plots for culture and biofluid samples separately (Fig. 3B and C), highlighting more clearly the impact of pellet and wash steps.

From the current study it would appear that ratios >3×10^10^ P/µg equate to high vesicular purity, ratios of 2×10^9^ to 2×10^10^ P/µg represent low purity, and any ratios below 1.5×10^9^ P/µg are unpure.

## Discussion

Here, we demonstrate a simple approach for assessing purity of single-source intra-laboratory vesicle preparations, which may also be beneficial across diverse biomarker, functional and future clinical studies.

The method clearly discriminates pure vesicle preparations from those replete with contaminating protein; proposing a ratio of 3×10^10^ particles per µg of protein, or greater as high purity. Preparations with lower ratios, around three times lower (1×10^10^ P/µg), can be achieved readily by simple pellet and wash protocols. These are naturally inferior purifications containing significantly higher levels of contaminating proteins. From the data shown, a decrease in ratio by 1×10^10^ P/µg can indicate an increase in contaminating proteins by 40% or more. This is an important consideration when selecting protocols for vesicular purification, which may have subsequent effects on analytical interpretation.

By performing these simple experiments, it was surprising how ineffective the wash step was at removing protein contaminants, providing as little as a 2-fold increase in ratio compared to the crude pellet. Whilst there is a consistent loss in total protein following ultracentrifugation based washing, there is also a loss in particle counts, due to incomplete recovery of available material. Hence, washing has only a small impact on elevating the ratio. Some of the proteins co-pelleted with vesicles at the first centrifugation remain present during the wash step, and may be simply co-purified with vesicles during the second step. If this is so, no amount of centrifugation-based washing will be effective in elevating the ratio of particles to protein. Approaches such as capturing vesicles based on their flotation characteristics, whilst more involved, reward by greater elimination of non-vesicular proteins. In the current study, purification by the sucrose-cushion method resulted in a ratio of 3.3×10^10^ P/µg. This was the highest ratio achieved in the study, suggesting that this was also the purest vesicular preparation.

Applying the ratio method to freshly collected biological fluids demonstrates the difficulties in reaching comparable purity as achievable with cultured cells. This, of course, is to be expected given the larger proteome of such source materials. However, these analyses raise major concerns about the purity of vesicles currently used in many studies. Using the pellet and wash protocol the final ratio achievable when dealing with biological fluids can be as low as 9.7×10^8^ P/µg. This is approximately 34-fold lower than the maximum purity that we were able to achieve using the sucrose-cushion method. From the spike in experiments, ratios below 2.5×10^9^ P/µg were evident when samples were >75% non-vesicular. Hence, we suggest that pellet and wash protocols for biological samples, achieving ratios of <2× 10^9^, lead to very poor purity samples.

Some alternative methods such as sucrose or OptiPrep^TM^ gradient centrifugation, dialysis, ultrafiltration or column chromatography may assist in elevating purity, above that achievable by the pellet and wash protocols, and potentially above that of the sucrose cushion approach. However, complex purification strategies like these are very time consuming and are unsuited to medium/high throughput analyses in relation to clinical trials (11) or biomarker exploration. Robust affinity capture based approaches are needed, but our method for purity assessment would likely be affected if this involves the addition of proteins such as antibodies.

In the analyses of biofluids, we were initially surprised to find that the ratio achieved with serum specimens was higher than that of urinary specimens. Given the very high level of protein in serum compared to urine, we had expected serum ratios to be strongly negatively impacted. However, this apparent discrepancy was due to the absolute concentration of particles in unpurified urine being very low, approximately 800-fold lower than that in serum. This is consistent with several studies requiring significant volumes of urine in order to generate useful quantities of exosomes (11,12), and accounts therefore for the low ratios seen in urine.

It is important to mention some caveats regarding the presented approach. Foremost is that the nanoparticle tracking approach cannot discriminate vesicles from non-vesicular particulate material; and here we have made the assumption that all detected particles are vesicles. This assumption may be unfair, as there may be protein aggregates, and large crystals of salts and other components present giving us an overestimation of the true number of vesicles present. We anticipate that as this technology platform evolves, particularly in relation to its capacity to measure fluorescent particles, future approaches will be able to discriminate vesicles from aggregated material, and aid in the refinement of our proposed method.

The other main caveat is that the method assumes that each vesicle has a comparable and stable quantity of protein. This aspect is again unlikely to be strictly true, as disease states may alter the protein content of vesicles somewhat (2). The ability of vesicles to passively interact with, and bind to various proteins in biological systems is currently underexplored.

Nevertheless, and bearing these issues in mind, the proposed method presents a useful and quantitative approach for establishing the purity of vesicle preparations, and highlights the likely need for additional purification strategies with respect to biological fluids that are required to improve vesicle-based biomarker and other studies.
